# Multidisciplinary tumor boards and their analyses: the yin and yang of outcome measures

**DOI:** 10.1186/s12885-021-07878-6

**Published:** 2021-02-17

**Authors:** Monika Engelhardt, Gabriele Ihorst, Martin Schumacher, Michael Rassner, Laura Gengenbach, Mandy Möller, Khalid Shoumariyeh, Jakob Neubauer, Juliane Farthmann, Georg Herget, Ralph Wäsch

**Affiliations:** 1 University of Freiburg Medical Center, Hematology & Oncology, Faculty of Medicine, Hugstetterstr. 53, 79106 Freiburg, Germany; 2 Comprehensive Cancer Center Freiburg (CCCF), Freiburg im Breisgau, Germany; 3 Clinical Trials Unit, Faculty of Medicine, Freiburg im Breisgau, Germany; 4 Medical Biometry and Statistics (IMBI), University of Freiburg, Faculty of Medicine, Freiburg im Breisgau, Germany; 5 Department of Radiology, Medical Center-University of Freiburg, Faculty of Medicine, University of Freiburg, Freiburg, Germany; 6 Department of Obstetrics and Gynecology, University Medical Center, Faculty of Medicine, Freiburg im Breisgau, Germany; 7 Department of Orthopaedics and Trauma Surgery, Medical Center, University of Freiburg, Faculty of Medicine, Freiburg, Germany

**Keywords:** Multidisciplinary tumor boards (MTBs), Outcome analysis, Adherence and compliance, Clinical trial inclusion, Satisfaction analysis

## Abstract

**Background:**

The standard to ensure utmost cancer treatment is a prerequisite in national cancer plans for comprehensive cancer centers (CCCs) and ensured through multidisciplinary tumor boards (MTBs). Despite these being compulsory for CCCs, various analyses on MTBs have been performed, since MTBs are resource-intensive. Outcome measures in these prior analyses had been survival (OS), MTB-adherence and -satisfaction, inclusion of patients into clinical trials and better cancer care.

**Main body:**

A publication from Freytag et al. performed an analysis in multiple tumor entities and assessed the effect of number of MTBs. By matched-pair analysis, they compared response and OS of patients, whose cases were discussed in MTBs vs. those that were not. The analysis included 454 patients and 66 different tumor types. Only patients with > 3 MTBs showed a significantly better OS than patients with no MTB meeting. Response to treatment, relapse free survival and time to progression were not found to be better, nor was there any difference for a specific tumor entity with vs. without MTB discussions.

An in-depth discussion of these results, with respect to the literature (PubMed search: “MTBs AND cancer”) and within the author group, including statisticians specialized in data analysis of cancer patients and questions addressed in MTBs, was performed to interpret these findings. We conclude that the results by Freytag et al. are deceiving due to an “immortal time bias” that requires more careful data interpretation.

**Conclusions:**

The result of Freytag et al. of a seemingly positive impact of higher number of MTBs needs to be interpreted cautiously: their presumed better OS in patients with > 3 MTB discussions is misleading, due to an immortal time bias. Here patients need to survive long enough to be discussed more often. Therefore, these results should not lead to the conclusion that more MTBs will “automatically” increase cancer patients’ OS, rather than that the insightful discussion, at best in MTBs and with statisticians, will generate meaningful advice, that is important for cancer patients.

## Background

The established standard to ensure state-of-the-art cancer treatment - as a prerequisite in the national cancer plan and for comprehensive cancer centers (CCCs) - is through multidisciplinary tumor boards (MTBs). Despite this being compulsory for outstanding cancer centers, various analyses on MTBs have been performed, because MTBs are resource-, personnel- and time-intensive [1]. Performed outcome measures had been better cancer care through interdisciplinary teams [2–5], improved response, progression free (PFS)- and overall survival (OS), enhanced adherence through electronically available MTB-protocols, higher patients’ and physicians’ satisfaction with cancer care [1, 6] and easier patient inclusion into clinical trials [1].

## Main text

A recent publication from Freytag et al. [7] challenges this notion as the authors assessed the effect of number of MTBs and potential differences between tumor entities. By matched-pair analysis, they compared response to treatment, OS, relapse or disease free survival and PFS of patients whose cases were discussed in MTBs vs. those that were not. The analysis was performed by the University of Bonn as a single center analysis between 2010 and 2016: after a matching process with a pool of 7262 patients, a total of 454 patients (6.3%), with as many as 66 different tumor types, were included in the study. Of interest, only patients with three or more (> 3) MTB meetings in their history showed a significantly better OS than patients with no MTB meeting. Moreover, response to treatment, relapse free survival and time to progression were not found to be better with vs. without MTB discussions. Moreover, there was no difference for a specific tumor entity with vs. without MTB discussions. The study concluded a positive impact of > 3 MTBs, whereas other positive outcome parameters remained negative [7].

Although we and others have demonstrated that with initiation of MTBs [1–5], patients discussed therein can be substantially increased, that MTB-questions mostly involve advice on best treatment, and that levels of compliance and evidence can be as high as > 90% [1], MTBs are resources-intensive. Advantages of MTBs are that they may improve patient inclusion into clinical trials and advance interdisciplinary projects [1, 6, 8]. In addition to the named MTB advantages, we had previously assessed the satisfaction of ~ 200 participants, referring physicians and patients, whose feed-back to MTBs had been exceedingly rewarding [1], thus encouraging cancer specialists to further engage themselves in them.

Median PFS and OS might even increase, although this is specifically challenging to verify, due to randomized MTB-trial designs being impossible to perform for ethical reasons. Moreover, MTB patients are often more difficult-to-treat and are referred to academic centers due to their complex disease, therefore questioning comparative analysis in their validity with prior non-MTB patients. Thus, matched-pair analysis may be inaccurate.

Freytag et al. show a time-lag-induced difference in OS in favor of those with > 3 MTB discussions for a small fraction of matched-paired patients with MTB vs. without. This is related to the fact, that patients needed to survive long enough for their cases to be discussed more often, and possibly also, because MTB physicians might care intensively in those with > 3 MTB discussions to obtain optimal cancer care.

In one prior analysis [1], we had assessed number of MTB discussions in multiple myeloma (MM) patients, which was - during our more condensed (2 years) time of assessment - mostly once (58%) or twice (26%), rather than 3- or > 4-times in 11 and 5%, respectively. Thus, repeated MTBs seem rarer than suggested by Freytag et al., revealed indeed more demanding to treat patients and suggest that shorter observation periods in one rather than multiple tumor entities are more meaningful [1].

In addition, the relatively limited patient number over the prolonged assessment period of 6 years in 66 different cancer entities [7] precluded from appropriate subgroup analyses, whereas in gynecological tumors, MTBs have shown impressive outcome results [2, 3]. That these cancer subgroups were entirely small can be attested, if 454 patients in 66 different cancers are divided to oversee approximate subgroup sizes (454: 66 = 7): this reveals exceedingly minute subgroups of different tumor entities (~ 7/subgroup/6 years = ~ 1/subgroup/year) [7].

Major other reasons contribute to the cardinal error of the analysis. Freytag et al. [7] found only 227 matching pairs, a wild mix of tumor entities from different MTBs being included, where it seemed to have been difficult to find matching partners. A statistical means to solve this would have been a dynamic matching process, in which patients are not further excluded (“error in matching”, Fig. 1) [7]. Matched pairs were selected based on at least 1 MTB in their history, thereby imposing a time restriction on the cases that was not made for the controls. Controls may die immediately after diagnosis, and this issue induces an immortal time bias. The Kaplan Meier curves reflect “time from diagnosis”, but the time aspect of MTBs was not considered in the statistical calculations. Although this is named in the discussion as survivorship bias (ostensibly because reviewers stated this as a weakness), this should have led to statistical refinements and exclusion of this time bias, rather than simply naming this phenomenon. Appropriate statistical approaches include landmark analyses or the incorporation of time-dependent covariates [9].

Another important aspect, also highlighted as a key weakness by the authors is the difference in follow-up time and therefore the exclusion of treatment evaluation over time as an influencing variable. It is left uncertain, how the division of patients diagnosed in 2011 vs. 2016 was generated over groups with 1–2 vs > 3 MTB discussions. This ignores a bias induced by gradually changing treatment options. As we could show in an analysis of anti-MM therapy, treatment options multiplied for cancer patients in just a couple of years [10]. Because of the missing focus on one tumor entity in this publication, important data, such as stage of disease or time of diagnosis at the point of inclusion into the study are not presented. Without these data possible confounders and reasons as to why a patient is discussed > 3 times in a MTB are missing. The reader is not able to distinguish, whether the frequency of discussion in a MTB was influenced by a) the possible difference in stages of the disease at diagnosis, b) growing number of treatment options or c) growing „popularity “of the use of MTBs in general [1].

Another disadvantage in the comparison of different tumor entities could be a different approach towards the use of MTB or even a standardized algorithm defining the time point and therefore the frequency of discussion within a MTB between tumor entities. While one entity might be curable through a defined treatment pathway or surgical intervention, without the need to further discuss treatment options, another may not be as easily curable with diverse treatment options [1, 10–12]. These entities are in need of an interdisciplinary discussion in varying frequencies, therefore confounding a possible comparability regarding OS.

Lastly, Freytag et al. did not shown, how many of the MTBs were done in patients after a relapse and how many of relapsed patients did receive only palliative care and no anti-tumor treatment. It is possible that more MTBs after relapse had an influence on OS; or vice versa, inclusion of more patients at initial diagnosis and with specific cancer diagnoses influenced OS. Thus, this article provides limited information, namely that: a) MTB consultations improved OS in only 74 patients, b) matched-pair analysis was performed in only <10% registered patients and c) immortal time bias was ignored for repetitive MTB-cases [7]. We conclude, that the analysis [7] needs to be interpreted with utmost caution and suggests that the number of MTBs does not make the difference, rather than the immortal time bias induced this error-prone survivorship advantage. Much sounder studies on MTBs have been generated [1–5] and serve as more valuable examples of interdisciplinary team efforts.

## Conclusions

Our conclusions are best expressed by citing a key sentence from the article by Anderson et al. [13]: “Analysis of survival by tumor response and similar analyses in which the primary outcome is compared among patients defined by some other outcome (dose-intensity, compliance to treatment, adverse event experience) using standard statistical approaches measuring outcome from the start of treatment are statistically invalid”. This and other important methodological articles have led to our correct interpretation of cancer care in close collaboration with statistical scientists [1, 14, 15], which is essential to produce reliable evidence for future progress. Remarkable in the Freytag publication [7] is finally that of the 19 authors, statisticians did not seem to have been involved.

We are delighted that at other centers, such productive and knowledgeable collaborations continue to exist [9].

## Data Availability

Not applicable.

## Abbreviations

MTBs: Multidisciplinary tumor boards
OS: Overall survival
CCCs: Comprehensive Cancer Centers
PFS: Progression free survival
MM: Multiple myeloma
